# Occurrence of Healthcare-Associated Infections (HAIs) by *Escherichia coli* and *Klebsiella* spp. Producing Extended-Spectrum β-lactamases (ESBL) and/or Carbapenemases in Portuguese Long-Term Care Facilities

**DOI:** 10.3390/pathogens11091019

**Published:** 2022-09-07

**Authors:** Elisabete Machado, Patrício Costa, Alexandre Carvalho, on behalf of the SAREL Investigators

**Affiliations:** 1 Local Coordination Group of the Program for the Prevention and Control of Infections and Antimicrobial Resistance (GCL-PPCIRA), Service of Quality, Safety and Epidemiology, Hospital de Braga, 4710-243 Braga, Portugal; 2 School of Medicine, University of Minho, 4710-057 Braga, Portugal; 3 Biomedical Research Center/UFP Energy, Environment and Health Research Unit (CEBIMED/FP-ENAS), University Fernando Pessoa, 4200-150 Porto, Portugal; 4 UCIBIO-Applied Molecular Biosciences Unit, Laboratory of Microbiology, Department of Biological Sciences, REQUIMTE, Faculty of Pharmacy, University of Porto, 4050-313 Porto, Portugal; 5 Life and Health Sciences Research Institute (ICVS), School of Medicine, University of Minho, 4710-057 Braga, Portugal; 6 ICVS/3B’s—PT Government Associate Laboratory, 4710-057 Braga/Guimarães, Portugal; 7 Service of Internal Medicine, Hospital de Braga, 4710-243 Braga, Portugal

**Keywords:** extended-spectrum β-lactam antibiotics, Enterobacterales, resistance, colonization, epidemiological surveillance

## Abstract

Extended-spectrum-β-lactamase (ESBL)- and carbapenemase-producing bacteria are widespread in hospitals, but the extent of this problem in long-term care facilities (LTCFs) is poorly understood. We aimed to elucidate, in the Portuguese regional clinical context, the relevance of LTCFs as a reservoir of *Escherichia coli* and *Klebsiella* spp. producing ESBL- and/or carbapenemases (Ec/Kp-ESBL/CARB). Fourteen LTCFs from Portugal, corresponding to units of convalescence (UC/*n* = 3), medium-term internment and rehabilitation (UMDR/ *n* = 5), or long-term internment and maintenance (ULDM/*n* = 6), were analyzed (2016–2019). All patients with Ec/Kp-ESBL/CARB infections acquired during LTCF stay were included, and detailed information was collected. Prevalence of patients with healthcare-associated infections (HAIs) by Ec/Kp-ESBL/CARB did not vary significantly over time (1.48% in 2016–2017, 1.89% in 2017–2018, and 1.90% in 2018–2019), but a statistically significant association with the LTCF typology (ULDM, UMDR) was observed. HAIs were caused by *K. pneumoniae* (*n* = 51/54.3%), *E. coli* (*n* = 41/43.6%), or both (*n* = 2/2.1%), producing ESBL (96%) or carbapenemases (4%). Prior colonization (*n* = 14/16%) corresponded to seven Kp-CARB and seven Ec/Kp-ESBL. The worrying prevalence of patients acquiring HAIs by Ec/Kp-ESBL/CARB, associated with the estimated rates of those already colonized at admission, highlights a relevant role for LTCFs as a reservoir of Ec/Kp-ESBL/CARB. Epidemiological surveillance should be extended to the national level, and colonization screening at LTCF admission implemented systematically.

## 1. Introduction

Antimicrobial resistance poses a serious threat to global health, leading to higher medical costs, longer hospital stays, and increased morbidity and mortality [1]. One of the main issues related to antimicrobial resistance is the exponential increase of infections caused by Enterobacterales (mainly *Klebsiella pneumoniae* and *Escherichia coli*) resistant to multiple antibiotics [1,2,3]. *Escherichia coli* and *Klebsiella pneumoniae* are common agents of different severe infections in hospitalized patients, and β-lactam antibiotics (mainly third-generation cephalosporins and carbapenems) have been the main therapeutic options [4]. However, their inadequate use has led to increased resistance rates to broad-spectrum β-lactams [1,3,4,5]. One of the main mechanisms of β-lactam resistance among *E. coli* and *Klebsiella* spp. is the production of extended-spectrum β-lactamases (ESBL) [6], which are currently spread among different clinically relevant Enterobacterales species, hosts, and ecological niches (hospitals and other healthcare institutions, the environment, animals, others) [5,7,8,9]. In infections involving ESBL producers, carbapenems are considered the therapeutic options of last resort [4,5,7], but during the last two decades several carbapenemases have reached global distribution [6,10]. Plasmids containing genes coding for ESBL and/or carbapenemases often also harbor other antimicrobial resistance genes, resulting in a depletion of therapeutic options [1,7,10].

Portugal is one of the European countries with the highest rates of resistance to third-generation cephalosporins and carbapenems among *E. coli* and *Klebsiella* spp. [3], with several studies characterizing the epidemiology of Enterobacterales producing ESBL (most recently of CTX-M type) and/or carbapenemases (especially KPC type) in hospitals [11,12,13,14,15]. However, in other healthcare institutions, the extent of this problem remains poorly understood [16,17,18,19].

Long-term care facilities (LTCFs) are becoming an increasingly important component of healthcare delivery systems. These inpatient units provide healthcare to people in a situation of dependence due to an acute illness or worsening of a chronic illness, with no need for hospital care, but requiring healthcare that cannot be provided at home [20]. Due to the very close relationship with hospitals (e.g., by the constant flow of patients between hospitals and LTCFs), many of the problems regarding healthcare-associated infections (HAIs) and colonization by multidrug-resistant bacteria, already known in hospitals, have begun to emerge and persist in LTCFs [16,17,19,21]. Currently, data on the occurrence and epidemiology of *E. coli* and *Klebsiella* spp. producing ESBL and/or carbapenemases in LTCFs in Portugal are scarce [16,17,19], and the role of LTCFs as a reservoir of these bacteria is unknown. Knowledge of the local, regional, national, and international epidemiology of these antibiotic-resistant bacteria is of added value for the optimization of antibiotic therapy [22] and for improving strategies to decrease their selection, persistence, and spread [23,24,25].

In this study, we investigated the occurrence of HAIs by ESBL- and/or carbapenemase-producing *Escherichia coli* and *Klebsiella* spp. acquired in LTCFs in Portugal over three years. We also elucidated, in the regional clinical context, the relevance of LTCFs as a reservoir of these strains.

## 2. Materials and Methods

### 2.1. Study Design and Patients

This was a retrospective, observational, cross-sectional, descriptive, and analytical study. 

The study included 14 internment LTCFs from the National Network of Long-Term Integrated Care [20], located in municipalities in the north of Portugal: Braga (*n* = 2), Fafe (*n* = 2), Felgueiras (*n* = 3), Guimarães (*n* = 3), Póvoa de Lanhoso (*n* = 1), and Vila Verde (*n* = 3). Units of convalescence (UC-A to -C, *n* = 3), units of medium-term internment and rehabilitation (UMDR-A to -E, *n* = 5), and units of long-term internment and maintenance (ULDM-A to -F, *n* = 6) were included [20]. Three one-year periods were analyzed, corresponding to August 2016 to July 2017 (2016–2017), August 2017 to July 2018 (2017–2018), and August 2018 to July 2019 (2018–2019). Table 1 shows some characteristics of the LTCFs analyzed.

All patients in whom *Escherichia coli* or *Klebsiella* spp. producing ESBL and/or carbapenemases were identified as etiological agents of infection, during the LTCF stay, were included. Patients whose signs and symptoms of infection appeared during the first 48 hours after LTCF admission, patients under 18 years of age, or whose clinical file had insufficient information were excluded from the study.

### 2.2. Data Collection

The identification of patients fulfilling the inclusion criteria in the study was performed by consulting the monthly records of patients under antibiotic therapy in each institution and the corresponding microbiological analysis results (bacterial identification and antibiogram), whenever a clinical sample was obtained for laboratory diagnosis of infection. For patients with multiple isolations of *E. coli* or *Klebsiella* spp. producing ESBL and/or carbapenemases, only those representing different antibiotic resistance phenotypes were considered.

Microbiological information (clinical sample and collection date, bacterial identification, antibiogram, information regarding a phenotype compatible with ESBL and/or carbapenemase production) was recorded for all patients included in the study. To collect sociodemographic and clinical data (gender, age, type of infection, clinical sample, antibiotic therapy, death during LTCF stay and reason, and if available, colonization by ESBL- and/or carbapenemase-producing *E. coli* or *Klebsiella* spp. at the time of LTCF admission), electronic medical records or file were used. Data were anonymized and stored in an encrypted document.

### 2.3. Statistical Analysis

Data were analyzed using Statistical Package for the Social Sciences (SPSS) Statistics^®^ software (version 25.0), provided by IBM (New Orchard Road, Armonk, New York 10504-1722, United States). Qualitative variables were presented as frequencies (*n*) and percentages (%). Quantitative variables were described using arithmetic mean (M) and standard deviation (SD). To compare the presence of *Escherichia coli* and *Klebsiella* spp. producing ESBL and/or carbapenemases by time, institution, and gender, the chi-square test (χ^2^) was used based on contingency tables. To analyze differences between categories, standardized residuals were compared (values greater than |1.96| were considered significant). *P* values below 0.05 were considered significant. Pearson *phi* (φ) or Cramer’s *V* coefficients were determined to assess the magnitude of the effect [26].

## 3. Results

### 3.1. Occurrence of HAIs by E. coli and/or Klebsiella spp. Producing ESBL and/or Carbapenemases

**Figure 1:**
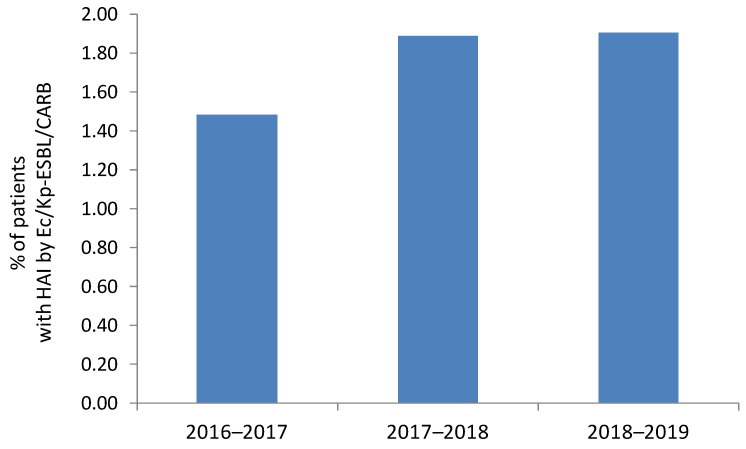
Prevalence of healthcare-associated infections (HAIs) by *E. coli* and/or *K. pneumoniae* producing ESBL or carbapenemases (Ec/Kp-ESBL/CARB) in the analyzed time periods.

The presence of ESBL- or carbapenemase-producing *E. coli* and/or *K. pneumoniae* (Ec/Kp-ESBL/CARB) as etiologic agents of at least one HAI was confirmed in 22 patients in 2016–2017, 31 patients in 2017–2018, and 34 patients in 2018–2019, corresponding to a prevalence of 1.48% (22/1484) in 2016–2017, 1.89% (31/1637) in 2017–2018, and 1.90% (34/1786) in 2018–2019 (Table 2, Figure 1). No statistically significant differences were found between the three periods, with a very small effect size (χ^2^(2, *n* = 4907) = 1.03, *p* = 0.597, Cramer’s *V* = 0.014).

In 8% (7/87) of patients, two HAI events by Ec/Kp-ESBL occurred during the LTCF stay. Simultaneous infection with ESBL-producing *E. coli* and *K. pneumoniae* was identified in 2.3% (2/87) of patients.

Patients who developed HAI by Ec/Kp-ESBL/CARB were aged between 48 and 96 years (M = 78.2; SD = 11.2), with 34.5% (30/87) being over 85 years old. We also found a slightly higher occurrence among men than among women: 2.00% (12/601) versus 1.13% (10/883) (2016–2017), 1.96% (12/611) versus 1.85% (19/1026) (2017–2018), and 2.36% (17/720) versus 1.59% (17/1066) (2018–2019). However, differences were not statistically significant, and the magnitude of the effect was very low (2016–2017: χ^2^(1, *n* = 1484) = 1.83, *p* = 0.176, φ = 0.035; 2017–2018: χ^2^(1, *n* = 1637) = 0.026, *p* = 0.872, φ = 0.004; 2018–2019: χ^2^(1, *n* = 1786) = 1.35, *p* = 0.245, φ = 0.028).

HAIs involving Ec/Kp-ESBL/CARB corresponded to urinary tract infections (UTIs) (91/94, 96.8%), tracheobronchitis (2/94, 2.1%), or surgical wound infections (1/94, 1.1%). Death occurred in six patients, the cause being HAI by ESBL-producing *K. pneumoniae* (*n* = 3; 3.4%; 1 ULDM/2016–2017; 1 UMDR/2017–2018; 1 ULDM/2018–2019) or another unspecified (*n* = 3; 3.4%; 2 ULDM, 1 UC).

### 3.2. Occurrence by LTCF Typology and Temporal Evolution by Institution

**Figure 2:**
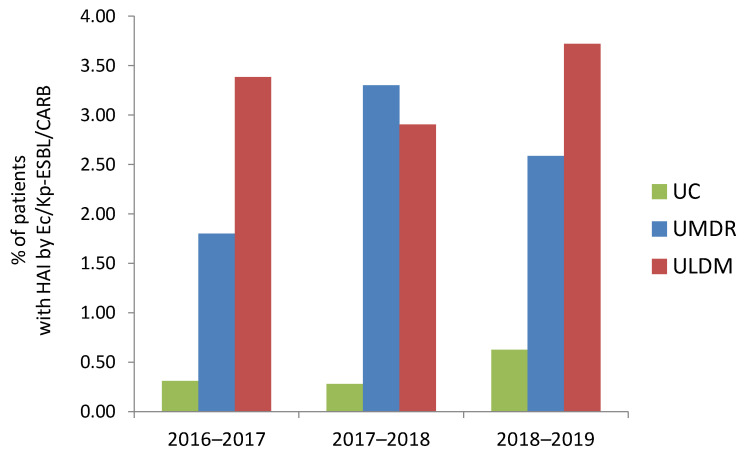
Prevalence of healthcare-associated infections (HAIs) by *E. coli* and/or *K. pneumoniae* producing ESBL or carbapenemases (Ec/Kp-ESBL/CARB) by LTCF typology, in the analyzed time periods. UC, units of convalescence (internments for up to 30 consecutive days); UMDR, units of medium-term internment and rehabilitation (internments between 30 and 90 consecutive days); ULDM, units of long-term internment and maintenance (internments of more than 90 days) [20].

Analysis of the occurrence of HAIs involving Ec/Kp-ESBL/CARB by LTCF typology revealed that it was consistently lower among patients at UC (0.30% in 2016–2017, 0.28% in 2017–2018 and 0.62% in 2018–2019) (Figure 2). In contrast, it was higher among patients at ULDM (especially in 2016–2017 and 2018–2019; 3.38% and 3.72%, respectively), or UMDR (especially in 2017–2018; 3.30%) (Figure 2). These differences were statistically significant, although the magnitude of the effect was either very low (2016–2017, 2018–2019) or low (2017–2018) (2016–2017: χ^2^(2, *n* = 1484) = 14.7, *p* = 0.001, Cramer’s *V* = 0.099; 2018–2019: χ^2^(2, *n* = 1786) = 14.4, *p* = 0.001, Cramer’s *V* = 0.090; 2017–2018: χ^2^(2, *n* = 1637) = 18.0, *p* ˂ 0.001, Cramer’s *V* = 0.105). Two HAI events were more frequent in ULDM (*n* = 5, 71.4%; four institutions) than in UMDR (*n* = 1) or UC (*n* = 1), with the bacteria involved in both events belonging to the same (*n* = 6; four *K. pneumoniae* and two *E. coli*) or different (*n* = 1) species. Simultaneous infection with ESBL-producing *E. coli* and *K. pneumoniae* (mentioned above) was only identified in patients at UMDR-C in 2017.

**Figure 3:**
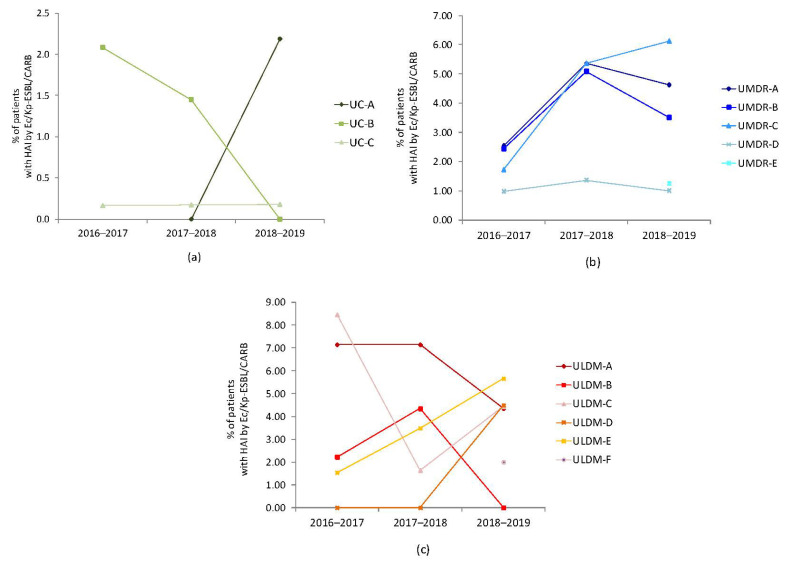
Temporal evolution of the occurrence of healthcare-associated infections (HAIs) by *E. coli* and/or *K. pneumoniae* producing ESBL or carbapenemases (Ec/Kp-ESBL/CARB) in each LTCF analyzed: (**a**) UC institutions (UC-A only started operating in 2018); (**b**) UMDR institutions (for UMDR-E only 2018–2019 data were available); (**c**) ULDM institutions (for ULDM-F only 2018–2019 data were available). UC, unit of convalescence (internments for up to 30 consecutive days); UMDR, unit of medium-term internment and rehabilitation (internments between 30 and 90 consecutive days); ULDM, unit of long-term internment and maintenance (internments of more than 90 days) [20].

Despite the lower occurrence of HAIs by Ec/Kp-ESBL/CARB among the UC typology, each institution’s temporal evolution was different (Figure 3a). Regarding UMDR, we observed an increase from 2016–2017 to 2017–2018, followed by a decrease during 2018–2019 (except for UMDR-C), with UMDR-D showing the lowest occurrence rates (Figure 3b). The temporal evolution of these infections in ULDM institutions was very diverse (Figure 3c).

### 3.3. Distribution by Etiological Agent and β-lactamase Type

**Figure 4:**
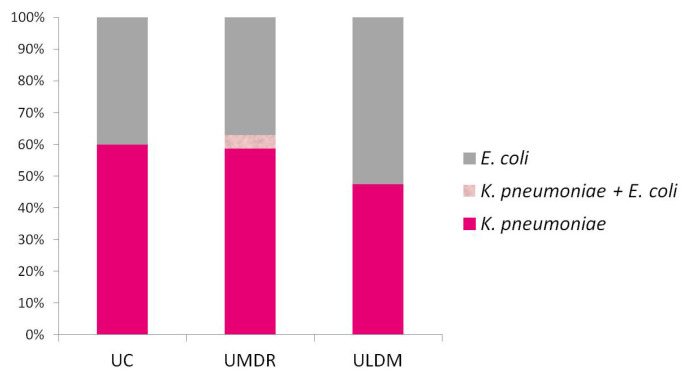
Distribution of etiological agents of the healthcare-associated infections (HAIs) analyzed in this study by LTCF typology. UC, units of convalescence (internments for up to 30 consecutive days); UMDR, units of medium-term internment and rehabilitation (internments between 30 and 90 consecutive days); ULDM, units of long-term internment and maintenance (internments of more than 90 days) [20].

**Figure 5:**
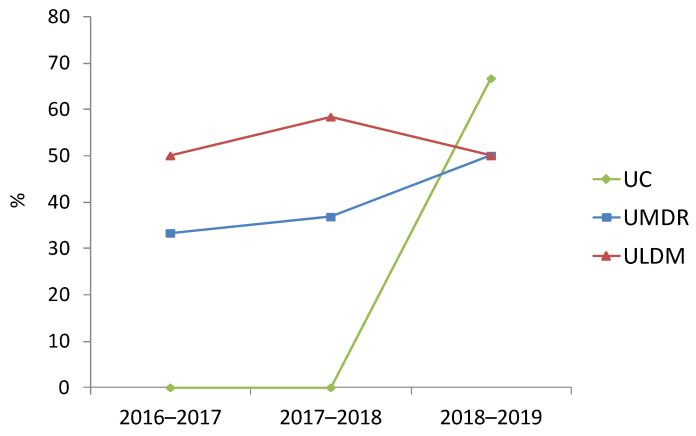
Temporal evolution of *E. coli* as an agent of the healthcare-associated infections (HAIs) analyzed in this study in the different LTCF typologies. UC, units of convalescence (internments for up to 30 consecutive days); UMDR, units of medium-term internment and rehabilitation (internments between 30 and 90 consecutive days); ULDM, units of long-term internment and maintenance (internments of more than 90 days) [20].

The etiological agents of HAIs in this study corresponded to *K. pneumoniae* (*n* = 51; 54.3%), *E. coli* (*n* = 41; 43.6%), or both (*n* = 2; 2.1%). *K. pneumoniae* was mainly found among patients in UC (*n* = 6; 60.0%) or UDMR (*n* = 27; 58.7%), whereas *E. coli* was more frequent in ULDM (*n* = 20; 52.6%) (Figure 4). A higher occurrence of *K. pneumoniae* was also observed in 2016–2017 (*n* = 15; 60.0%) and 2017–2018 (*n* = 19; 57.6%), whereas *E. coli* was the most frequent (*n* = 19; 52.8%) in 2018–2019. To understand whether this recent increase in *E. coli* was related to its increase among patients at ULDM (the typology with the highest rate of HAIs by *E. coli*), we analyzed the temporal evolution of HAIs by *E. coli* in the three LTCF typologies. We found that this recent increase in the relative frequency of *E. coli* infections did not occur in ULDM (*n* = 7/58.3% in 2017–2018 and *n* = 6/50% in 2018–2019), but rather in UC and UMDR (UC: *n* = 0/0.0% in 2017–2018 and *n* = 4/66.7% in 2018–2019; UMDR: *n* = 7/33.8% in 2017–2018 and *n* = 9/50% in 2018–2019) (Figure 5).

ESBL producers were involved in 96% (90/94) of the HAIs, corresponding to *K. pneumoniae* (*n* = 48), *E. coli* (*n* = 40), or *K. pneumoniae* and *E. coli* (*n* = 2). ESBL types (TEM, SHV, CTX-M, others) were not mentioned in the microbiological results. Carbapenemase producers (4%) were identified as *K. pneumoniae* (*n* = 3; 1 UC-C/2018–2019, 1 UMDR-A/2016–2017, and 1 ULDM-E/2018–2019) or *E. coli* (*n* = 1; 1 UMDR-A/2017–2018), with carbapenemase type (KPC) being reported for two *K. pneumoniae* (Table 2).

### 3.4. Co-Resistance to Non-β-lactam Antibiotics

Ec/Kp-ESBL/CARB isolates involved in the HAIs analyzed in this study were frequently co-resistant to norfloxacin (46/47, 97.9%), levofloxacin (22/23, 95.7%), ciprofloxacin (69/74, 93.2%), gentamicin (71/89, 79.8%), and trimethoprim-sulfamethoxazole (67/96, 69.8%). Co-resistance to fosfomycin (21/90, 23.3%), nitrofurantoin (17/85, 20%), or amikacin (0/31, 0%) was observed less frequently.

### 3.5. Antibiotic Therapy Implemented and Clinical Outcome

In most HAIs by Ec/Kp-ESBL/CARB, clinical sample collection was followed by empirical therapy further adjusted according to the microbiological results, although in others the antibiogram was awaited to start targeted therapy. Nitrofurantoin, fosfomycin, and trimethoprim-sulfamethoxazole were the most prescribed antibiotics, accounting for more than 60% of prescriptions for these infections (mostly UTIs). Carbapenems were administered to three patients who were admitted to a hospital for carbapenem treatment.

The clinical outcome was cure in 93% of patients. However, 3.4% (*n* = 3) died due to the infection, including one (1.1%) with an ESBL-producing *K. pneumoniae* and under therapy with cefuroxime, a cephalosporin highly hydrolyzed by ESBL.

### 3.6. Colonization Prior to LTCF Admission

Patients acquiring HAIs by Ec/Kp-ESBL/CARB during their LTCF stay came directly from a hospital (*n* = 43; 49.4%), from another LTCF (*n* = 38; 43.7%), or from the household (*n* = 6, 6.9%). Assessment of intestinal colonization by Ec/Kp-ESBL/CARB at LTCF admission was not routinely performed, despite one LTCF starting, in September 2018, the systematic screening of intestinal colonization by carbapenemase producers at patient ingress. Hence, previous colonization status was mainly analyzed by consulting the clinical information sent by the patient’s home institution, which only allowed us to estimate it. The clinical information of 14 patients (16%) mentioned the need for contact isolation upon LTCF admission, due to colonization with carbapenemase-producing *K. pneumoniae* (*n* = 7; 4 KPC; 3 UMDR, 2 ULDM, 2 UC), ESBL-producing *K. pneumoniae* (*n* = 4; 2 UMDR, 1 ULDM, 1 UC), or ESBL-producing *E. coli* (*n* = 3; 2 ULDM, 1 UMDR). Colonization of the urinary and/or intestinal tract (*n* = 11; both species) was the most frequent, followed by pressure-ulcer wounds (*n* = 2, *E. coli*) or the respiratory tract (*n* = 1, *K. pneumoniae*). For 71.4% (*n* = 5) of the patients previously colonized with ESBL producers and 28.6% (*n* = 2) colonized with carbapenemase producers, there was a diagnosis of HAI by ESBL- or KPC-producing bacteria, respectively, from the same species (probably the colonizing strain). The remaining patients colonized with carbapenemase producers developed HAIs by ESBL-producing bacteria of the same (*n* = 3; 42.8%) or different (*n* = 2; 28.6%) colonizing species. In 73 patients, previous colonization was unknown at the time of LTCF admission.

## 4. Discussion

Long-term care facilities are one of the three levels of healthcare (alongside hospital and primary healthcare), functioning as an interface between the hospital and the community [20]. However, LTCF patients often come from hospitals and with characteristics similar to the hospitalized population. Therefore, some well-known problems in the hospital environment, such as HAIs involving multidrug-resistant microorganisms, are also arising in LTCFs [27]. Several international and national studies have reported the prevalence of patients with HAIs acquired in LTCFs [28,29,30,31,32]. In the HALT studies (*Healthcare-Associated Infections in European Long-Term Care Facilities*), promoted by the European Centre for Disease Prevention and Control (ECDC), point prevalence values of 2.4% (2010), 3.4% (2013), and 3.1% (2016–2017) were reported in Europe, with data for Portugal corresponding to 7.4% (2010), 9.5% (2013), and 4.3% (2016–2017) [28,33,34]. Since the classification of institutions providing long-term care varies greatly from country to country, institutions representing the reality of LTCFs in Portugal were not considered in these studies [20]. Therefore, the Portuguese Directorate-General of Health carried out three national studies [32], revealing prevalence rates above that published in European reports: 8.1% (2012), 10.4% (2013), and 6.8% (2017). However, no studies have analyzed the prevalence of HAIs acquired in LTCFs by Enterobacterales producing ESBL or carbapenemases, which currently pose major challenges to antibiotic therapy, especially considering the emergence of pandrug-resistant strains [2].

This study analyzed, for the first time in Portugal, the prevalence of HAIs by ESBL- or carbapenemase-producing *E. coli* and/or *Klebsiella* spp. acquired in LTCFs, revealing worrying prevalence rates, which might still be underestimated by the number of HAIs clinically diagnosed. According to the ECDC, collection of clinical samples to guide therapy occurs in about 59.4% of antibiotic prescriptions at LTCFs in Portugal (25% in Europe) [28,32,34]. Urinary tract infections (UTIs) were the most frequent (96.8%) HAIs, but these data should be interpreted with caution, especially considering the apparent low accessibility of LTCFs to microbiological analysis and the ease of urine collection and processing [34], as well as the national and international studies reporting similar relative distributions of different HAI types [28,32,33,34]. No association was found between the acquisition of HAIs (mostly UTIs) and gender, probably because of the advanced age of patients (M = 78.2; SD = 11.2) (incidence of UTI over age 65 generally does not differ much between genders).

No significant differences were found in the prevalence of these infections among the three time periods. Therefore, there does not seem to be a trend for its increase over time, which may be related to the strengthening of measures included in the guidelines of the Program for the Prevention and Control of Infections and Antimicrobial Resistance (PPCIRA) implemented in our country [32].

In the three time periods, a statistically significant association was observed between the acquisition of HAI by Ec/Kp-ESBL/CARB and LTCF typology. The highest prevalence among patients at ULDM or UMDR was in agreement with national studies analyzing the prevalence of patients with infections in LTCFs [32]. Risk factors such as reduced mobility, urinary and/or fecal incontinence, wounds or pressure ulcers, and age over 85 years have been associated with ULDM (and, to a lesser extent, UMDR) patients [32] and may justify this finding. The occurrence of two HAI events (*n* = 5; 71.4%) or death from infection (*n* = 2; 66.7%) was also more common among ULDM patients. Each institution presented a particular temporal evolution, probably reflecting characteristics of the admitted population and/or specific institution-time practices for the prevention and control of infections and antimicrobial resistance. In fact, this diverse temporal evolution might be related to the type of population that each LTCF was receiving during the time analyzed and, therefore, to the upstream problems of LTCF (the hospitals or other healthcare institutions-of-origin of the patients). The number of patients entering the LTCF coming from healthcare institutions with or without intrinsic epidemiological problems with ESBL and/or carbapenemase producers, might influence the number of patients with colonization and/or HAI by these bacteria in the LTCF analyzed. This is even more relevant if we take into account that studies have reported high rates of ESBL-producing *K. pneumoniae* and *E. coli*, and increasing rates of carbapenemase-producing *K. pneumoniae* in Portuguese hospitals, including hospitals located in the same geographic area considered in this study [11,12,13,14,15]. Even so, specific institutional practices for the prevention and control of infections and antimicrobial resistance may also have contributed to those observations.

The higher occurrence of HAIs by ESBL- or carbapenemase-producing *K. pneumoniae* until 2017–2018 was in accordance with the epidemiological changes observed in these periods in Portugal, namely the increase of ESBL-producing *K. pneumoniae* and the emergence of carbapenemase-producing *K. pneumoniae* in hospitals, following the epidemiological trends described in other countries [13,15,35,36]. Corroborating these data, studies have reported high rates of intestinal colonization by ESBL-producing *K. pneumoniae* among patients at LTCFs, most belonging to clones circulating in hospitals in the same geographic area considered in this study, as well as infections by *K. pneumoniae* producing KPC-3 in LTCFs [16,17,19]. However, for 2018–2019 we observed higher rates of HAIs by *E. coli*, mainly through its increase in UC and UMDR. The recent ECDC report does not support this observation [3]. Future research should clarify whether this alteration corresponds to a real epidemiological change (e.g., driven by successful pandemic clones as ST131) or whether it is biased by the low number of HAIs with laboratory diagnosis in LTCFs.

## 5. Conclusions

This study reveals a worrying prevalence of patients with HAIs by ESBL- or carbapenemase-producing *E. coli* and/or *K. pneumoniae* at LTCFs, and reinforces the persistence and spread of these bacteria in healthcare institutions in Portugal, an epidemiological trend also reported in other countries [3,9,10]. Together with the estimated rates of patients already colonized at admission, it highlights, in the regional clinical context, the relevant role of LTCFs as a reservoir of ESBL- or carbapenemase-producing *E. coli* and/or *K. pneumoniae*.

This epidemiological situation represents a major challenge not only for patient safety and quality of care, but also for public health, imposing the need for better access to microbiological analysis, rigorous practices of infection prevention and control, and improved antibiotic stewardship [22,27,37,38,39,40]. Epidemiological prospective surveillance of HAIs by ESBL or carbapenemase producers in LTCFs at the national level, as well as systematic screening of colonization by these bacteria in LTCFs and other healthcare institutions, is imperative to improve local, regional, national, and international strategies for prevention and control of their spread.

## Figures and Tables

**Table 1:** Characterization of the long-term care facilities (LTCFs) included in the study (2016–2019).

Typology *^a^*	Institution	Geographic Location	Number of beds	Number of Interned Patients								
				August 2016–July 2017			August 2017–July 2018			August 2018–July 2019		
				TOTAL (*n* = 1484)	F (*n* = 883)	M (*n* = 601)	TOTAL (*n* = 1637)	F (*n* = 1026)	M (*n* = 611)	TOTAL (*n* = 1786)	F (*n* = 1066)	M (*n* = 720)
UC	UC-A	Municipality VI	17	**NA**	NA	NA	**67 *^b^***	50	17	**183**	122	61
	UC-B	Municipality III	14	**48**	26	22	**69**	42	27	**62**	36	26
	UC-C	Municipality V	28	**611**	376	235	**580**	391	189	**561**	362	199
UMDR	UMDR-A	Municipality VI	33	**197**	114	83	**168**	93	75	**173**	102	71
	UMDR-B	Municipality III	18	**41 *^c^***	30	11	**59**	39	20	**57**	29	28
	UMDR-C	Municipality II	10	**58**	36	22	**56**	33	23	**49**	30	19
	UMDR-D	Municipality IV	30	**204 *^d^***	124	80	**293**	170	123	**298**	178	120
	UMDR-E	Municipality I	24	**ND**	ND	ND	**ND**	ND	ND	**80 *^e^***	40	40
ULDM	ULDM-A	Municipality VI	33	**42**	21	21	**56**	29	27	**46**	21	25
	ULDM-B	Municipality III	32	**45 *^c^***	28	17	**46**	29	17	**40**	25	15
	ULDM-C	Municipality II	22 *^f^*	**71**	39	32	**61**	37	24	**45**	22	23
	ULDM-D	Municipality IV	30	**102**	50	52	**96**	60	36	**89**	57	32
	ULDM-E	Municipality IV	35	**65**	39	26	**86**	53	33	**53**	25	28
	ULDM-F	Municipality I	24	**ND**	ND	ND	**ND**	ND	ND	**50**	17	33

Abbreviations: F, female; M, male; NA, not applicable (LTCF did not exist during this period); ND, not determined (it was not possible to access the necessary data). *^a^* Typology of the internment LTCF: UC, unit of convalescence (internments for up to 30 consecutive days); UMDR, unit of medium-term internment and rehabilitation (internments between 30 and 90 consecutive days); ULDM, unit of long-term internment and maintenance (internments of more than 90 days) [20]. *^b^* The LTCF started operating in early 2018, so this number refers to the period March 2018–July 2018. *^c^* Data available only for the period February 2017–July 2017. *^d^* The LTCF only became operational in the second half of 2016, so this number refers to the period September 2016–July 2017. *^e^* Data available only for the period January 2019–July 2019. *^f^* In April 2019, this LTCF started to provide 34 beds for internment.

**Table 2:** Distribution of patients with at least one healthcare-associated infection (HAI) by *E. coli* and/or *K. pneumoniae* producing ESBL or carbapenemases (Ec/Kp-ESBL/CARB) by different periods, LTCF typology, and institution.

Typology *^a^*	Institution	Number of Patients with HAI by Ec/Kp-ESBL/CARB			TOTAL
		2016–2017 (*n* = 22)	2017–2018 (*n* = 31)	2018–2019 (*n* = 34)	
UC	UC-A	NA	0	4	4
	UC-B	1	1	0	2
	UC-C	1	1	1 *^b^*	3
	***Total***	***2***	***2***	***5***	***9***
UMDR	UMDR-A	5 *^b^*	9 *^b^*	8	22
	UMDR-B	1	3	2	6
	UMDR-C	1	3	3	7
	UMDR-D	2	4	3	9
	UMDR-E	ND	ND	1	1
	***Total***	***9***	***19***	***17***	***45***
ULDM	ULDM-A	3	4	2	9
	ULDM-B	1	2	0	3
	ULDM-C	6	1	2	9
	ULDM-D	0	0	4	4
	ULDM-E	1	3	3 *^b^*	7
	ULDM-F	ND	ND	1	1
	***Total***	***11***	***10***	***12***	***33***

Abbreviations: NA, not applicable (LTCF still did not exist in this period); ND, not determined (data unavailable for this period). *^a^* Typology of the internment LTCF: UC, unit of convalescence (internments for up to 30 consecutive days); UMDR, unit of medium-term internment and rehabilitation (internments between 30 and 90 consecutive days); ULDM, unit of long-term internment and maintenance (internments of more than 90 days) [20]. *^b^* One patient with HAI by carbapenemase-producing bacteria.

## Data Availability

Not applicable.
